# CAL-1 as Cellular Model System to Study CCR7-Guided Human Dendritic Cell Migration

**DOI:** 10.3389/fimmu.2021.702453

**Published:** 2021-09-16

**Authors:** Edith Uetz-von Allmen, Guerric P. B. Samson, Vladimir Purvanov, Takahiro Maeda, Daniel F. Legler

**Affiliations:** ^1^Biotechnology Institute Thurgau (BITg) at the University of Konstanz, Kreuzlingen, Switzerland; ^2^Graduate School for Cellular and Biomedical Sciences (GCB), University of Bern, Bern, Switzerland; ^3^Department of Laboratory Medicine, Nagasaki University Graduate School of Biomedical Sciences, Nagasaki, Japan; ^4^Theodor Kocher Institute, University of Bern, Bern, Switzerland; ^5^Department of Biology, University of Konstanz, Konstanz, Germany

**Keywords:** human dendritic cell line, cell migration, chemokine receptor CCR7, CCL19, CCL21, chemotaxis, expression of fluorescent reporter proteins, CRISPR/Cas9 mediated CCR7 knockout

## Abstract

Dendritic cells (DCs) are potent and versatile professional antigen-presenting cells and central for the induction of adaptive immunity. The ability to migrate and transport peripherally acquired antigens to draining lymph nodes for subsequent cognate T cell priming is a key feature of DCs. Consequently, DC-based immunotherapies are used to elicit tumor-antigen specific T cell responses in cancer patients. Understanding chemokine-guided DC migration is critical to explore DCs as cellular vaccines for immunotherapeutic approaches. Currently, research is hampered by the lack of appropriate human cellular model systems to effectively study spatio-temporal signaling and CCR7-driven migration of human DCs. Here, we report that the previously established human neoplastic cell line CAL-1 expresses the human DC surface antigens CD11c and HLA-DR together with co-stimulatory molecules. Importantly, if exposed for three days to GM-CSF, CAL-1 cells induce the endogenous expression of the chemokine receptor CCR7 upon encountering the clinically approved TLR7/8 agonist Resiquimod R848 and readily migrate along chemokine gradients. Further, we demonstrate that CAL-1 cells can be genetically modified to express fluorescent (GFP)-tagged reporter proteins to study and visualize signaling or can be gene-edited using CRISPR/Cas9. Hence, we herein present the human CAL-1 cell line as versatile and valuable cellular model system to effectively study human DC migration and signaling.

## Introduction

Dendritic cells (DCs) are sentinels of the innate and adaptive immune system and play essential roles in initiating, coordinating and regulating adaptive immune responses (1). They reside in peripheral tissues where they are poised to capture and process antigens derived from invading pathogens. Upon pathogen encounter, DCs undergo a complex process of maturation, induce the expression of co-stimulatory molecules and migrate *via* the lymph system to the next draining lymph node (2, 3). In lymph nodes, DCs present pathogen-derived antigens to cognate T cells to trigger an adaptive immune response (4). Due to the unique ability of DCs to prime and activate naïve T cells, DC-based vaccination strategies are exploited as cancer therapies in which DCs are loaded with tumor antigens to mount tumor antigen specific immune responses (5–7). Notably, DCs comprise a heterogeneous population of cells (8–10). Human peripheral blood includes two main DC populations, namely myeloid and plasmacytoid DCs, with different functional properties (11). Nonetheless, one key feature for their use as an effective cellular vaccine is that the tumor antigen loaded DCs must efficiently migrate to lymphoid organs to encounter and prime tumor antigen specific T cells (7). Leukocyte migration is controlled by the expression of specialized chemokine receptors and integrins (12). Importantly, expression of the chemokine receptor CCR7 is essential to all DC subtypes for their homing to lymphoid organs (3). In fact, DCs up-regulate the expression of CCR7 upon encounter of a danger signal and acquire a migratory phenotype (13, 14). CCR7-expressing DCs migrate along CCL19 and CCL21 chemokine gradients and *via* the lymph system to reach the T cell zone of the next draining lymph node (15, 16). Besides controlling DC homing, CCR7 also coordinates the recruitment of circulating T cells from the blood to the lymph nodes and acts as co-stimulatory molecule to efficiently prime T cells (17). Notably, besides common expression by interstitial lymphatic endothelial cells and lymphoid tissue stroma cells (18), disparate lymphoid chemokine expression has been noted between men and mice. CCL21 is not produced, but trapped and presented by human high endothelial venules (HEVs), whereas in mice this chemokine is synthesized directly by HEVs (19).

CCR7-dependent DC migration has extensively been studied using *in vitro* differentiated bone-marrow derived precursor cells, so called BMDCs, obtained from wild-type or gene targeted mice as cellular model systems. Recent technical advances, although not trivial, enabled the transient immortalization of murine hematopoietic precursor cells by a retrovirally delivered and induced expression of the transcription factor Hoxb8 (20). Upon relieve from the induced Hoxb8 expression, such precursor cells can be differentiated in the presence of GM-CSF to immature DCs that resemble immature BMDCs (21, 22). However, the usefulness of murine BMDC cultures in the study of DC biology has been debated (23, 24). Moreover, the technical achievement of using Hoxb8-DCs are currently restricted to mouse DC models. Human DC subsets can be isolated in very limited numbers from peripheral blood (11) and hence are not exploited as cellular model systems to study molecular mechanisms of DC migration. The most widely used human DC model system is monocyte-derived DCs (MoDCs), which comprises the isolation of peripheral blood monocytes and their *in vitro* differentiation to MoDCs in the presence of GM-CSF and IL-4 for several days (7, 25–28). A drawback of these MoDCs is that they show substantial donor to donor variation, are refractory to genetic manipulation and hence not suitable as effective cellular model to study CCR7-driven DC migration. Attempts to use human leukemia cell lines with monocyte-like properties under DC differentiating culture conditions were of limited success (29). Own attempts to induce endogenous CCR7 expression in any of these human cell lines failed. A DC-like cell line of human origin that upon exposure to a danger signal induces CCR7 expression and migration combined with the ability to be genetically edited is lacking but highly desired to study human DC migration.

A human plasmacytoid DC line, termed CAL-1, has been established from a patient with blastic natural killer cell lymphoma (30), also referred to as blastic plasmacytoid DC neoplasm (31). CAL-1 cells were shown to express transcripts for TLR2, TLR4, TLR7 and TLR9 and are sensitive to single-stranded RNA (a ligand for TLR7), to Resiquimod R848 (R848; a ligand for TLR7/8) and to unmethylated CpG oligodeoxynucleotides (ODNs; ligands for TLR9) (30, 32, 33). Moreover, upon TLR ligation, CAL-1 cells are reported to secrete the cytokines TNFα, IFNγ, and IL-6, and to express the co-stimulatory molecules CD40, CD80 and CD86 (30, 32). However, unlike plasmacytoid DCs, CAL-1 cells respond to IL-3 and GM-CSF to form dendrites, a characteristic feature of MoDCs, and up-regulate MHC class II (30, 34). Here, we demonstrate that CAL-1 cells exposed to GM-CSF up-regulate the expression of CCR7 in response to a danger signal. Moreover, we show that these cells are well suited to study CCR7 signaling and cell migration. Finally, we provide evidence that CAL-1 cells can be genetically modified – e.g. to express a fluorescent reporter protein – while retaining their migratory capacity in response to the chemokines CCL19 and CCL21 in 2D and 3D environments.

## Materials And Methods

### Cell Culture

CAL-1 cells, obtained under a material transfer agreement, were cultured in complete growth medium RPMI 1640 containing 2mM L-alanyl-L-glutamine (Pan Biotech Ref. P04-18500, Chemie Brunschwig, Basel, Switzerland), supplemented with 10% FCS (Gibco Ref. 10270-106; LuBioScience, Luzern, Switzerland) and 1% penicillin/streptomycin (BioWhittaker Lonza; VWR Scientific, Nyon, Switzerland) in suspension tissue culture flasks or plates (Greiner Bio-One; Huberlab, Aesch, Switzerland). Floating cells were passaged to new culture flasks for cell maintenance. Where indicated, CAL-1 cells (2x 10^5^ cells/ml) were exposed to 10ng/ml human GM-CSF (Peprotech, LuBioScience; catalog #300-03) for 3 days, referred to as GM/CAL-1 cells, washed, resuspended at a density of 1x 10^6^ cells/ml in complete growth medium without GM-CSF and then left untreated or matured for another 18-19h with either the TLR7/8 agonist Resiquimod R848 (10μg/ml; Sigma–Aldrich, Buchs, Switzerland; #SML0196) or the TLR9 ligand CpG-B ODN 2006 (1μM; InvivoGen; LabForce, Muttenz, Switzerland; #tlrl-2006-1).

### Flow Cytometry

Cultured cells were collected, washed and resuspended in complete growth medium at 1x 10^6^ cells/ml and stained for 20min at 4°C using the following anti-human antibodies (1:20): APC-labeled CCR7 and the matched isotype control antibody (R&D Systems; Bio-Techne, Zug, Switzerland), CD123 (clone 6H6, Abeomics; LucernaChem, Luzern, Switzerland), CD11c (clone 3.9), HLA-DR (BioLegend through LucernaChem), and FITC-labeled anti-CD40 (Serotec; Bio-Rad, Cressier, Switzerland) or anti-CD86 (BD Biosciences, Allschwil, Switzerland). Unbound antibodies were removed by washing with 1x PBS and cell pellets were resuspended in FACS buffer (1x PBS supplemented with 0.5% FCS). Samples were filtered (50μM Cup Filcons from BD), measured with a BD LSRII or a LSRFortessa flow cytometer using the BD FACSDiva™ 6 software and analyzed with the FlowJo software (BD). SYTOX^®^ Blue or TO-PRO™-3 iodide (Invitrogen; Thermo Fisher Scientific, Allschwil, Switzerland) was added as a dead cell indicator.

### Generation of CAL-1 Cell (sub) Lines Stably Expressing the PH-Akt-GFP Reporter

CAL-1 cells were transfected with 5μg of the plasmid DNA pcDNA3-PH-Akt-EGFP (35) using the Neon™ Transfection System 100µl Kit with buffer E2 (Invitrogen; Thermo Fisher). Cells (1x 10^6^) were electroporated using a single pulse of 1600V and 20ms after resuspension in Opti-MEM^®^ I reduced serum medium (Gibco; Thermo Fisher). The transfected cells (24h) were grown under a selective pressure of 0.3mg/ml G418 for two weeks and then bulk sorted for medium to high GFP expression on a BD Aria IIu cell sorter (BD Biosciences). Aliquots of this CAL-1 PH-Akt-GFP^line^ were frozen. Experiments with the CAL-1 PH-Akt-GFP^line^ were performed 19-26 days after bulk sorting (that is 34-41 days after transfection) with one freeze-thaw cycle in between. A frozen sample of the CAL-1 PH-Akt-GFP^line^ was thawed, cultured for 11 days under continuous G418 selection and used to establish two sub-lines (CAL-1 PH-Akt-GFP^subline 1^ and CAL-1 PH-Akt-GFP^subline 2^) by cell sorting. Aliquots of the sublines were frozen 6 days after sorting. Experiments with CAL-1 PH-Akt-GFP^subline 1^ or CAL-1 PH-Akt-GFP^subline 2^ were performed 7-10 days, respectively 8-15 days, after thawing.

### Generation of CCR7 Knockout and Parental CAL-1 Cell Clones

CRISPR/Cas9–based gene knockout (KO) CAL-1 clones lacking the CCR7 gene were established using the U6-gRNA : CMV-Cas9-2a-tGFP transfection (p01) plasmid from Sigma and the Neon transfection system (5μg DNA per 1x 10^6^ cells; one pulse of 1600V and 20ms). The single-guide RNA (sgRNA)–targeting sequence in the CCR7 gene was 5′-CGCAACTTTGAGCGCAACA (CRISPRD HSPD0000007879). Single cell clones were FACS-sorted into 96-well tissue culture plates 48h after transfection based on GFP expression (0.5% of the cell population) using a BD Aria IIu cell sorter and cultured under standard maintenance conditions. Colony formation was observed under a microscope. Due to the lack of a specific antibody to CCR7 that would work in Western blotting, site-specific genome editing was verified using PCR or PCR combined with blunt end cloning followed by Sanger sequencing. Additional cell clones of the parental CAL-1 cell line were established by single cell sorting using a BD Aria IIu cell sorter. Three individual clones were randomly selected, expanded and subsequently differentiated and matured. Chemokine binding and cell migration experiments of these three cell clones were performed 27-29 days after single cell sorting.

### FITC-Dextran Uptake Assay

CAL-1 or GM/CAL-1 cells (2x 10^6^) were stimulated with 1µg/ml Resiquimod R848 for 30min before incubating with 1mg/ml FITC-dextran (FD40, Sigma-Aldrich) for the indicated time points at 37°C. Alternatively, CAL-1 or GM/CAL-1 cells were matured with 1µg/ml Resiquimod R848 or 1μM CpG-B ODN 2006 for 24h before cells were incubated with FITC-dextran for 30min. The reaction was stopped by adding ice-cold RPMI medium. Then, cells were extensively washed with ice-cold medium and once with PBS before being resuspended in FACS buffer (1x PBS, 2% FCS). FITC-dextran uptake kinetics was quantified by flow cytometry.

### Intracellular Cytokine Staining (ICS)

CAL-1 or GM/CAL-1 cells (2x 10^5^ in 180μl medium) were incubated in the presence or absence of TLR ligands (1μM CpG-B or 1μg/ml R848) in a 96 well round bottom plate for 6h at 37°C, 5% CO_2_. Brefeldin A (10μg/ml) was added for the last 4h of stimulation. Cells were centrifuged for 5min at 300x g, fixed for 20min on ice using 4% PFA and then washed twice with 200μl permeabilization buffer (2mM EDTA, 2% FCS, 0.1% saponin, 0.02% NaN_3_ in 1x PBS). Intracellular cytokine production was measured by flow cytometry using the following staining: cells were incubated with APC conjugated anti-human TNFα (clone Mab11, BioLegend, 1:20) for 90min on ice in the presence of permeabilization buffer, then washed with permeabilization buffer and resuspended with ice-cold FACS buffer.

### Cytosolic Free Ca^2+^ Mobilization

R848-matured GM/CAL-1 cells (1x 10^6^/ml) were loaded with 4μM fluo-3-AM (Molecular Probes/Invitrogen; Thermo Fisher) in loading-buffer (145mM NaCl, 5mM KCl, 1mM Na_2_HPO_4_, 1mM MgCl_2_, 5mM glucose, 1mM CaCl_2_, and 10mM HEPES, pH 7.5) for 20min at 37°C and then washed twice with loading-buffer. Where indicated, R848-matured GM/CAL-1 cells were pre-treated with 200ng/ml of *Bordetella pertussis* toxin (PTx; BML-G100, Enzo Life Sciences, Lausen, Switzerland) for 3h prior to fluo-3 loading. Human chemokine-induced cytosolic free calcium mobilization-related fluorescence intensity changes were recorded over time by flow cytometry as described (36). Data were corrected for mean basal fluorescence intensity before stimulation and normalized to the mean intensity in the presence of ionomycin (1μg/ml). Flow cytometry analysis was performed in parallel to ensure CCR7 surface expression.

### Cell Stimulation and Western Blot Analysis

R848-matured GM/CAL-1 cells were washed with RPMI 1640 medium without additives (RPMI) and incubated at a density of 1x 10^6^ cells/ml in serum-free RPMI for 2h at 37°C, 5% CO_2_. Serum starved cells were scraped and washed once with RPMI. Aliquots of 1.5x 10^6^ cells in 50μl RPMI were incubated for 5min at 37°C and then stimulated with 50μl of pre-warmed RPMI or medium containing a 2-fold chemokine concentration (final concentration: 100nM of human CCL19 or CCL21, which is known to induce maximal responses (37, 38); Peprotech #300-29B and #300-35) for indicated time points. Cell stimulation was terminated by the addition of 25μl of 5x Laemmli sodium dodecyl sulphate (SDS) loading buffer containing 4% β-mercaptoethanol. DNA was immediately sheared by vortexing or rapidly passing the sample up and down through a 200μl tip several times. Samples were boiled and proteins (15μl whole cell lysates) were separated under reducing conditions on 10% SDS-polyacrylamide gel electrophoresis (SDS-PAGE) and analyzed by Western blot (39, 40) using the respective antibodies from Cell Signaling (BioConcept, Allschwil, Switzerland), diluted 1:1’000 in staining buffer (1x PBS, 3% BSA, 0.05% Tween 20, 0.02% NaN_3_) and rolling overnight at 4°C: rabbit anti-p44/42 MAPK (t-Erk1/2), mouse anti-phospho-p44/42 MAPK (Thr202/Tyr204; p-Erk1/2) and rabbit anti-phospho-Akt (Ser473; p-Akt) after blocking the membranes for 1h at RT with 1x Roti^®^-Block (Carl Roth, Arlesheim, Switzerland). A mAb against β-actin was used as loading control (Abcam; LucernaChem). After washing with PBS-T buffer (1x PBS, 0.02% Tween 20), HRP-conjugated secondary antibodies diluted 1:5’000 in PBS-T buffer containing 5% low-fat dry milk were bound and detected using Clarity™ Western ECL Substrate (Bio-Rad). Thereafter, membranes were stripped with Restore™ Western Blot Stripping Buffer (Thermo Fisher) for 15min at RT and then washed and re-probed with β-actin control antibody. Band volume intensities for p-Erk1/2 or p-Akt were quantified using the Image Lab Software Version 4.1 (Bio-Rad) and normalized to t-Erk. A portion of R848-matured GM/CAL-1 cells was analyzed for CCR7 surface expression by flow cytometry for each experiment.

### Chemokine Binding Assay

Individual GM/CAL-1 CCR7-KO clones, GM/CAL-1 control cells and cell clones were matured with Resiquimod R848 and assessed for fluorescent CCL19-S6^649P1^ (41) binding at 7°C. A 96 well V-plate containing 90µl per well of a 2x 10^6^ per ml cell suspension (in RPMI) was equilibrated at 7°C for 15min. Ten µl of a 200nM chemokine solution (final chemokine concentration: 20nM) or RPMI medium alone were added and incubated with the cells for 30min. The plate was centrifuged for 3min at 10°C, the supernatant discarded and the cells washed once with 150µl ice cold RPMI followed by a washing step with FACS buffer. CCL19-S6^649P1^ fluorescence was measured on a BD LSRFortessa flow cytometer.

### 2D-Transwell Migration Assay

R848-matured GM/CAL-1 cells, stable transfected (sub)lines, or gene edited GM/CAL-1 cell clones (1×10^5^ in complete growth medium) were seeded into the top chambers on a polycarbonate filter with a pore size of 5μm in a 24-well Transwell plate (Corning Costar; Vitaris, Baar, Switzerland) and allowed to migrate towards the lower chamber wells containing 600μl of complete growth medium without chemokine (random migration) or supplied with 30nM human CCL19 or CCL21, an optimal concentration for DCs in this assay (37, 38), for 3h at 37°C, 5% CO_2_. A 500μl aliquot of migrated cells was collected and acquired for 60sec at high flow rate on a BD LSRII or for 90sec at medium flow rate on a BD LSRFortessa flow cytometer (39). Values are given as percentage of migrated cells relative to the input of cells.

### 3D Migration Assay and Imaging

Migration through a three-dimensional collagen type I gel matrix was performed in tissue culture-treated ibiTreat µ-slide chemotaxis chambers (ibidi, Vitaris, Baar, Switzerland) as described (42, 43). Briefly, dead cells were removed using a dead cell removal kit, and the remaining viable R848-matured GM/CAL-1 cells, cell clones or stable transfected (sub)lines were resuspended at 10^7^ cells/ml in RPMI supplemented with 10% heat inactivated FCS. Twenty μl 10x DMEM, 10μl 7.5% NaHCO_3_, and 150μl PureCol collagen I (Advanced Biomatrix; CellSystems, Troisdorf, Germany) were premixed, carefully mixed with 90µl cell suspension, applied to µ-slide chemotaxis chambers, and then allowed to polymerize. Chemokines were added to the right reservoir (100nM) and allowed to establish a stable gradient as described (42). Cell migration was recorded using the Zeiss ZEN pro 2012 software by time-lapse video microscopy at 2-min intervals using a 10x objective and a MRm camera on a Zeiss Axiovert 200M equipped with an automated stage and a 37°C environmental Tokai Hit INU (Shizuoka Japan) incubation system for 2.5h (75 frames). Cell tracking was performed using the ImageJ/Fiji (NIH, National Institutes of Health, USA) plugin ‘Manual Tracking’ and migration parameters were quantified using the ‘Chemotaxis and Migration Tool’ from ibidi. Analysis was restricted to motile cells by eliminating objects that displaced less than twice the cell body size during the 2.5h imaging process. For live cell imaging, migrating R848-matured GM/CAL-1 cells stably expressing PH-Akt-GFP were imaged on a Zeiss Axiovert 200M (5min intervals) and on a laser scanning microscope (Leica TCS SP5; Leica, Heerbrugg, Switzerland using the HCX PL APO CS 63.0×1.40 OIL UV objective) at 1min intervals using a microscope stage fitted with a Tokai Hit Thermoplate at 37°C.

### Statistical Data Analysis

Data are presented in the figures as means ± SEM or SD, as indicated in the corresponding figure legends. For multiple group comparisons, a one‐way ANOVA test was performed, followed by Dunnett’s or Tukey’s multiple comparisons tests using GraphPad Prism 6.07 (GraphPad Software, San Diego, CA, USA). Asterisks in graphs indicate statistical significance (****p < 0.0001; ***p < 0.001; **p < 0.01; *p < 0.05).

## Results

### Phenotypic Characterization of Human CAL-1 Cells

**Figure 1:**
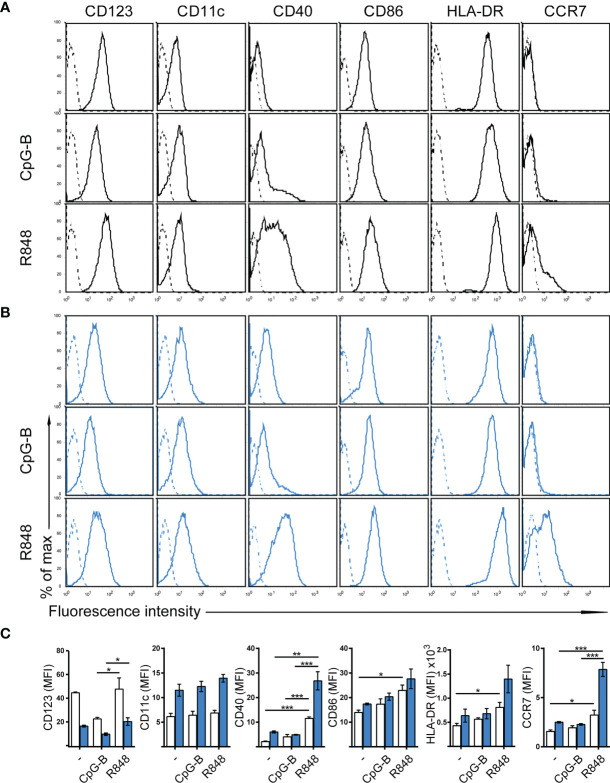
Phenotypic characterization of human CAL-1 cells and CCR7 induction upon exposure to GM-CSF and maturation by R848. **(A)** Surface expression of CD123, CD11c, CD40, CD86, HLA-DR and CCR7 (solid lines) on CAL-1 cells that were stimulated or not for 18-19h with the TLR9 ligand CpG-B ODN 2006 (1μM) or the TLR7/8 ligand Resiquimod R848 (10μg/ml). Representative flow cytometry histograms derived from one out of three independent experiments are depicted. Unstained cells or isotype-matched controls for CCR7 stainings are shown as dashed lines. **(B)** Surface expression of CD123, CD11c, CD40, CD86, HLA-DR and CCR7 on CAL-1 cells cultured for 3 days in the presence of 10ng/ml GM-CSF. Where indicated, these GM/CAL-1 cells were in addition matured by either CpG-B or R848 as in **(A)**. Representative flow cytometry histograms derived from one out of three independent experiments are depicted. **(C)** Quantitative analysis of surface markers on CAL-1 (white bars) and GM/CAL-1 (blue bars) cells. Mean values ± SEM of the 3 independent experiments **(A, B)** are shown.

The human dendritic cell line CAL-1 originates from a patient with blastic natural killer cell lymphoma (30), a disease also known as blastic plasmacytoid dendritic cell neoplasma (31). Accordingly, CAL-1 cells were reported to express the human pDC marker CD123 (IL-3Rα), together with the MHC class II molecule HLA-DR and low levels of the α_x_ integrin CD11c (30). Using flow cytometric analysis, we confirm that CAL-1 cells expressed CD11c and high levels of CD123 and HLA-DR (**Figure 1A**). Moreover, CAL-1 cells also expressed the co-stimulatory molecule CD86, but barely expressed the co-stimulatory molecule CD40 (**Figure 1A**). The chemokine receptor CCR7, required for lymph node homing of DCs, was not detected on the surface of CAL-1 cells (**Figure 1A**). Exposing CAL-1 cells to the danger signal and DC maturation stimulus CpG-B (a ligand for TLR9) for 18h did not substantially alter CD123, CD11c, CD86 or HLA-DR expression and failed to effectively induce surface expression of CD40 and CCR7 (**Figure 1A**). Similarly, maturing CAL-1 cells with the TLR7/8 ligand R848, although inducing CD40, only marginally induced CCR7 on some of the cells (**Figure 1A**).

### CCR7 Induction in CAL-1 Cells Upon Exposure to GM-CSF and Maturation by R848

CAL-1 cells were reported to enhance CD11c surface expression (30) and to up-regulate MHC class II RNA expression (34) upon cultivation in the presence of GM-CSF for 3 days. Hence, we exposed CAL-1 cells for 3 days to GM-CSF and subsequently induced maturation by either CpG-B or R848 for 18 to 19h. Notably, CAL-1 cells cultured for 3 days in GM-CSF, subsequently termed GM/CAL-1 cells, expressed lower levels of CD123 (**Figures 1B, C** blue bars) compared to CAL-1 cells cultured in the absence of GM-CSF (**Figures 1A, C** white bars), irrespectively whether cells were in addition matured by CpG-B or R848 (**Figure 1**). By contrast, GM/CAL-1 cells expressed slightly higher surface levels of CD11c than CAL-1 cells cultured under traditional conditions, and CD11c expression levels remained unaltered upon maturation (**Figures 1B** **, C**). The expression of the co-stimulatory molecules CD40 and CD86, as well as HLA-DR, were highest in GM/CAL-1 cells matured with R848 (**Figures 1B** **, C**). Importantly, GM/CAL-1 cells matured by R848, but not by CpG-B, profoundly induced surface expression of CCR7 (**Figures 1B** **, C**), a prerequisite for the homing of antigen-bearing DCs to draining lymph nodes. Collectively these results indicate that the human dendritic cell line CAL-1 acquires a more myeloid-like phenotype if exposed to GM-CSF, and most importantly up-regulates the expression of the chemokine receptor CCR7 upon maturation by the TLR7/8 ligand R848.

### FITC-Dextran Uptake and TNFα Production by CAL-1 and GM/CAL-1 Cells

**Figure 2:**
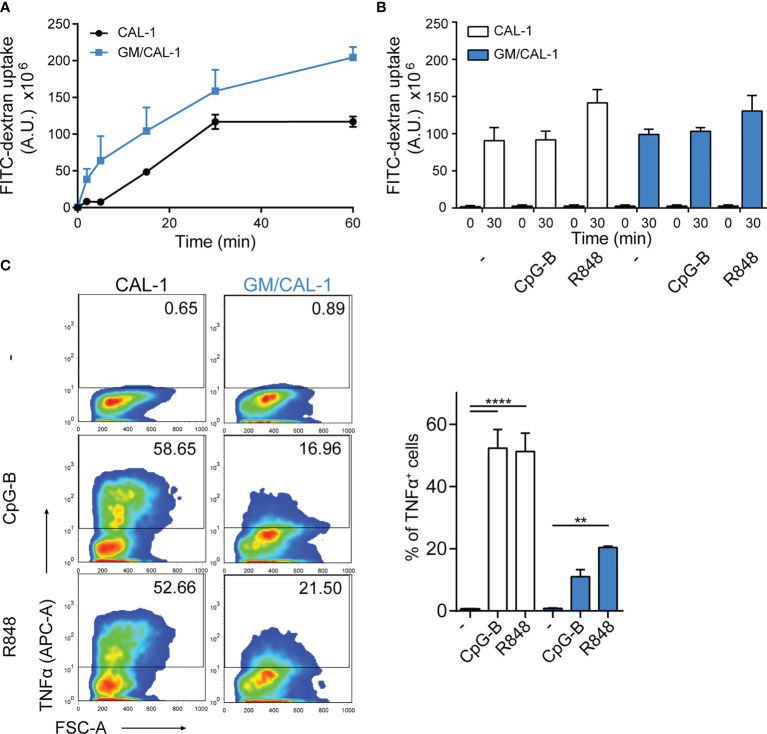
FITC-dextran uptake and TNFα production by CAL-1 and GM/CAL-1 cells **(A)** Time-dependent FITC-dextran uptake by CAL-1 and GM/CAL-1 cells. CAL-1 cells were cultured in the absence (CAL-1; back line) or presence of GM-CSF (GM/CAL-1; blue line) for 3 days, pre-stimulated with R848 for 30min and subsequently incubated with 1mg/ml of FITC-dextran for indicated time periods at 37°C. Cells were extensively washed and FITC-dextran uptake was quantified by flow cytometry. The area under the curve [A.U.] was calculated by multiplying the mean fluorescence intensity (MFI) values and the event count of live, FITC-positive cells. Mean values ± SEM of 3 independent experiments are shown. **(B)** FITC-dextran uptake by mature CAL-1 and GM/CAL-1 cells. CAL-1 (white bars) and GM/CAL-1 (blue bars) were left untreated (–) or matured with either CpG-B or R848 for 24h. FITC-dextran uptake at t=0 or t=30min of incubation with 1mg/ml of FITC-dextran was determined and quantified by flow cytometry as in **(A)**. Mean values ± SEM of 3 independent experiments are shown. **(C)** TNFα production by CAL-1 and GM/CAL-1 cells. CAL-1 or GM/CAL-1 cells were stimulated or not with CpG-B or R848 for 6h. For the last 4h of incubation 10μg/ml Brefeldin A was added to prevent cytokine secretion. Cells were fixed, permeabilized, and the percentage of cells with intracellularly accumulated TNFα was determined by ICS and flow cytometry. One representative pseudocolor dot plot (left panels) and quantification of three (bar graphs on the right panel; CAL-1: white bars, GM/CAL-1: blue bars) independent experiments are shown as mean values ± SEM.

Next, we determined FITC-dextran uptake as a surrogate for antigen uptake by these human cells. For this, we pre-stimulated CAL-1 cells with R848 for 30min and determined FITC-dextran uptake over time by flow cytometry. As shown in **Figure 2A**, CAL-1 cells readily and continuously took up FITC-dextran. Similarly, R848 pre-stimulated GM/CAL-1 cells retained their capacity to take-up FITC-dextran (**Figure 2A**). Maturing CAL-1 and GM/CAL-1 cells with either CpG-B or R848 for 24h before adding FITC-dextran for 30min barely affected the cell’s capacity to take-up FITC-dextran (**Figure 2B**). As expected (32, 33), stimulating CAL-1 cells with either CpG-B or R848 strongly induced the production of TNFα as determined by a flow cytometric intracellular cytokine staining assay (**Figure 2C**). GM/CAL-1 cells also produced TNFα upon TLR stimulation (**Figure 2C**), however, the percentage of cytokine producing cells was reduced compared to CAL-1 cells cultured in the absence of GM-CSF.

### Mature GM/CAL-1 Cells Elicit CCR7 Signaling Pathways

**Figure 3:**
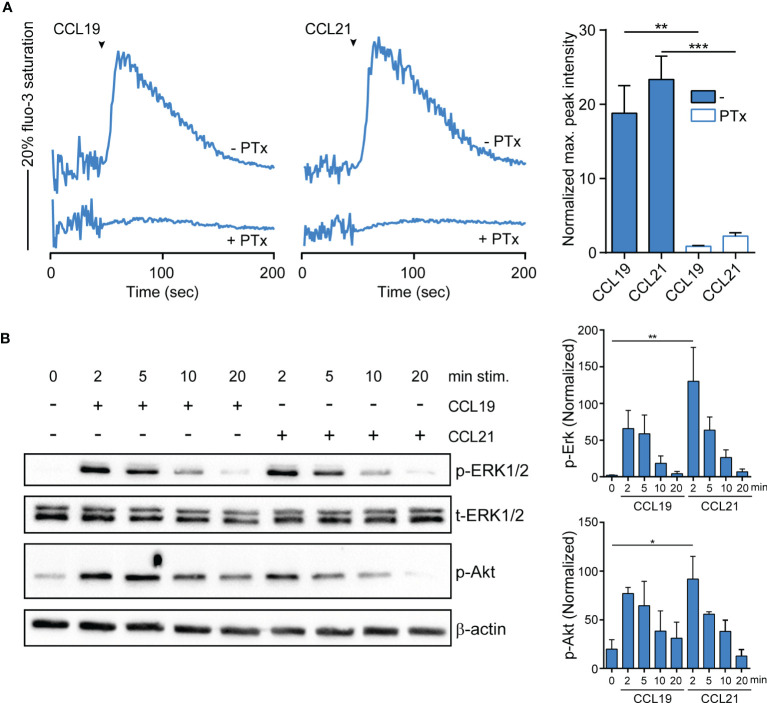
CCR7-mediated calcium mobilization and phosphorylation of ERK1/2 and Akt in R848-matured GM/CAL-1 cells. **(A)** CCL19/CCL21-induced calcium mobilization in mature GM/CAL-1 cells. R848-matured GM/CAL-1 cells were treated or not with 200ng/ml PTx for 3h, loaded with fluo-3-AM and stimulated with 30nM of either CCL19 or CCL21. Changes in intracellular calcium levels were recorded by flow cytometry over time. Chemokine addition is indicated by arrowheads. One representative (left panels) and mean values ± SEM of 3 independent experiments (right panel) are shown. Maximal peak intensity of chemokine-induced fluo-3 fluorescence was baseline corrected and normalized to ionomycin treatment by calculating the mean of ten maximal fluo-3 fluorescence values derived from each stimulation condition, respectively. **(B)** CCR7 triggering results in transient ERK1/2 and Akt phosphorylation. R848-matured GM/CAL-1 cells were stimulated with 100nM of either CCL19 or CCL21 for the indicated time periods. Chemokine-mediated activation of ERK1/2 and Akt was determined by Western blotting. Phosphorylation was determined using antibodies recognizing the phosphorylated forms of Thr202/Tyr204 of ERK1/2 (p-ERK1/2), and the phosphorylated form of S473 of Akt (p-Akt). Antibodies recognizing total ERK1/2 (t-ERK) and β-actin served as control for equal protein loading. One representative (left panel) and mean values ± SEM of 3 independent experiments (right panel) are shown.

To assess whether R848-driven GM/CAL-1 cell maturation induces functional CCR7 expression, we determined if chemokine stimulation elicits signal transduction. Activation of chemokine receptors by cognate ligands typically mobilize intracellular calcium (44), which is required for human MoDC migration (45). Stimulating R848-matured GM/CAL-1 cells with 30nM of either CCL19 or CCL21 induced a rapid and transient increase in the cytosolic free Ca^2+^ ([Ca^2+^]_i_) (**Figure 3A**). Chemokine-mediated [Ca^2+^]_i_ mobilization was abrogated by *Bordetella pertussis* toxin (PTx) treatment (**Figure 3A**), which manifests G_i_-protein dependent CCR7 signaling. Furthermore, we determined additional signaling pathways downstream of CCR7. Both ligands induced the phosphorylation of ERK1/2 of the MAP-kinase signaling cascade in R848-matured GM/CAL-1 cells (**Figure 3B**). Chemokine-mediated ERK1/2 phosphorylation peaked at 2min after CCR7 triggering and remained detectable for up to at least 10min. Similarly, CCL19 and CCL21 stimulation resulted in transient phosphorylation of protein kinase B (PKB)/Akt (**Figure 3B**).

### Mature GM/CAL-1 Cells Readily Migrate Towards CCR7 Ligands

**Figure 4:**
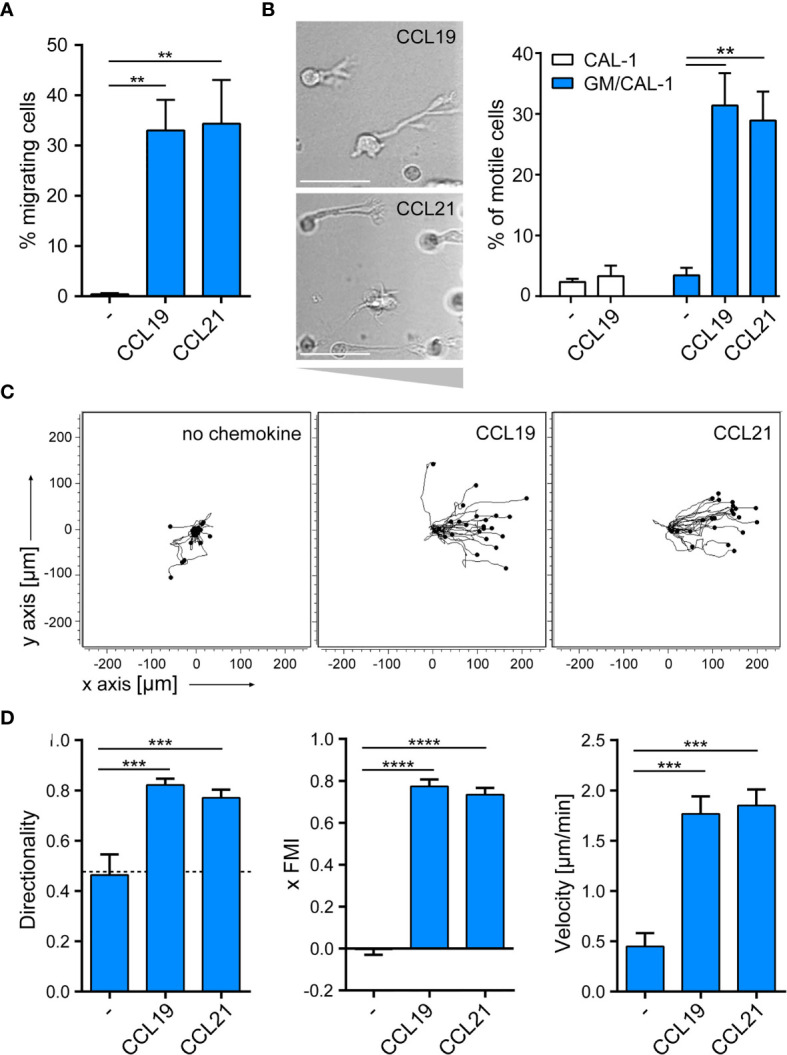
R848-matured GM/CAL-1 cells readily migrate towards CCR7 ligands in 2D and 3D environments. **(A)** Mature GM/CAL-1 cells specifically migrate in response to CCL19 and CCL21 in 2D Transwell migration assays. CAL-1 cells were cultured for 3 days in 10ng/ml GM-CSF followed by maturation with 10μg/ml R848 for another 18.5h. R848-matured GM/CAL-1 cells were allowed to migrate towards medium (–), or 30nM of either CCL19 or CCL21 in Transwell chemotaxis chambers for 3h. Percent migrated cells was determined by flow cytometry. Mean values ± SD derived from n = 3 independent experiments are shown. **(B–D)** Mature GM/CAL-1 cells migrate directionally along chemokine gradients in 3D. R848-matured GM/CAL-1 cells were embedded into a three-dimensional collagen type I gel matrix in Ibidi µ-slide chemotaxis chambers and allowed to migrate along CCL19 or CCL21 gradients. Cell migration was monitored by time-lapse video microscopy (time lag τ = 2 min). **(B)** Representative DIC images of migrating mature GM/CAL-1 cells are shown on the left panel. Scale bar: 50μm. Percentage of motile CAL-1 (not treated with GM-CSF; white bars) or GM/CAL-1 (blue bars) cells in a 3D environment in the presence or absence of chemokine derived from all cells located in the entire matrix area is depicted on the right. Mean values ± SEM of at least 3 independent experiments are shown. **(C)** Single R848-matured GM/CAL-1 cells randomly moving in the absence (left panel), or migrating towards CCL19 (middle panel) or CCL21 (right panel panel) from a representative experiment were tracked using an ImageJ/Fiji plug-in and individual tracks are shown in a spider plot. **(D)** Directionality, xFMI and velocity of individually migrating cells derived from three independent experiments were quantified. Mean values ± SEM of 3-6 independent experiments are shown. At least 10 cells per condition for every experiment, and a total of 65, 112 or 92 cells were analyzed for steady state (in the absence of chemokine), CCL19- or CCL21- directed migration, respectively.

Next, we assessed the migratory capacity of R848-matured GM/CAL-1 cells in two-dimensional Transwell chemotaxis assays where cells migrate across a semipermeable filter into the lower compartment containing the chemokine. Mature GM/CAL-1 cells essentially did not migrate towards medium, but effectively migrated in response to 30nM of either CCL19 or CCL21 (**Figure 4A**). Subsequently, we monitored cell migration by time-lapse video microscopy where we embedded mature GM/CAL-1 cells in a collagen I three-dimensional gel matrix within ibiTreat µ-slide chemotaxis chambers. R848-matured GM/CAL-1 cells exposed to CCL19 or CCL21 gradients established within this 3D-device showed a polarized phenotype typical for migrating cells (**Figure 4B**). Notably, about 30% of the R848-matured GM/CAL-1 cells exposed to either CCR7 ligand were motile; i.e. displaced more than twice the cell diameter within the 2.5h period of measurement (**Figure 4B**). In the absence of chemokines, less than 4% of the R848-matured GM/CAL-1 cells were motile (**Figure 4B**). For comparison, as little as 2.4 ± 0.5% of R848-matured CAL-1 cells (i.e. that were not exposed to GM-CSF) were motile and only marginally more cells, namely 3.3 ± 1.7%, were motile if stimulated with CCL19 (**Figure 4B**). By contrast, R848-matured GM/CAL-1 cells efficiently and readily migrated along CCL19 and CCL21 gradients in a 3D collagen environment (**Figure 4C**). Tracking the paths of migrating cells revealed a directionality of R848-matured GM/CAL-1 cells of 0.82 ± 0.03 for CCL19, 0.77 ± 0.03 for CCL21, and 0.46 ± 0.08 for medium, respectively (**Figure 4D**). The forward migration indices on the x-axis along the chemokine gradient (xFMI) were 0.77 ± 0.03 for cells migrating towards CCL19 and 0.73 ± 0.03 for cell migration in response to CCL21, whereas the xFMI of cells migrating in response to medium was -0.00 ± 0.03 (**Figure 4D**). Migrating R848-matured GM/CAL-1 cells reached a velocity of 1.77 ± 0.17 μm/min in the direction of CCL19 and 1.85 ± 0.16 μm/min towards CCL21, but only 0.45 ± 0.13 μm/min in response to medium (**Figure 4D**). Hence, we demonstrated that GM/CAL-1 cells matured by R848 express functional CCR7, elicit early chemokine-mediated G_i_-dependent signaling pathways and readily migrate in response to chemokines in 2D and 3D environments.

### Mature GM/CAL-1 Cells Serve as a Versatile Model System to Study Human DC Migration

**Figure 5:**
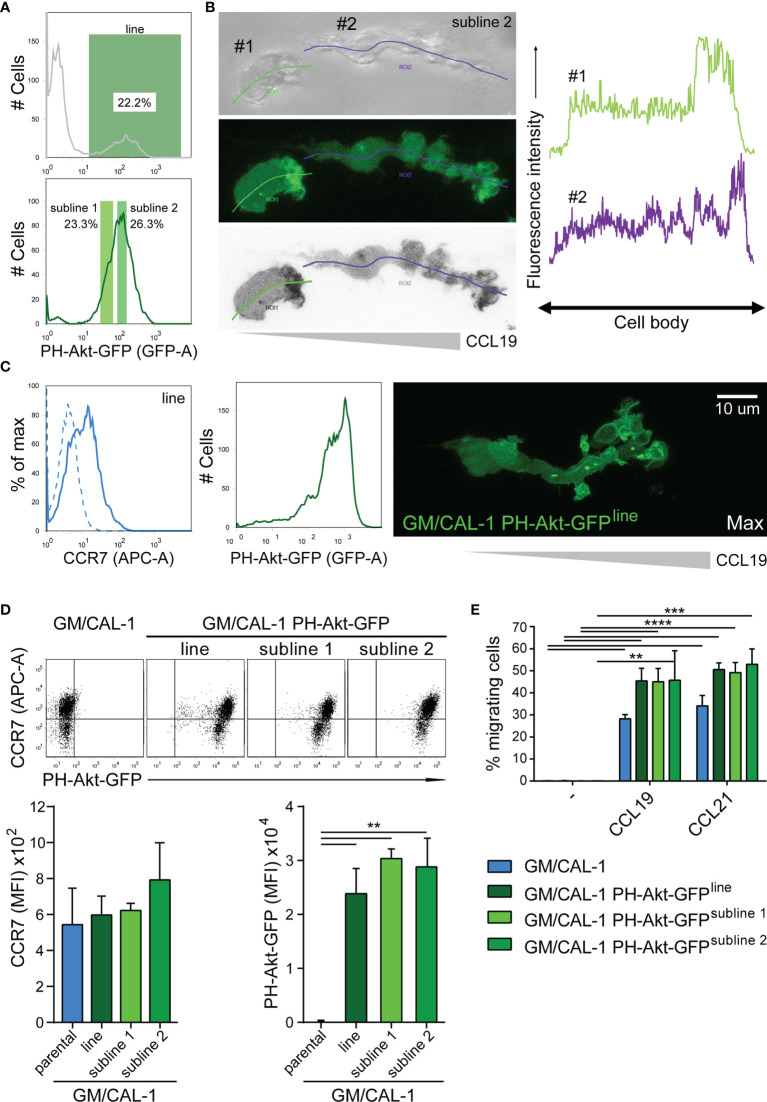
Establishing stable cell lines to monitor PH-Akt-GFP, a biosensor for PIP_3_, in migrating R848-matured GM/CAL-1 cells. **(A)** Generation of a stable CAL-1 PH-Akt-GFP line and two sublines. CAL-1 cells were nucleofected with a plasmid coding for PH-Akt-GFP. After two weeks of selection, 22.2% of the cell population stably expressed PH-Akt-GFP and was bulk sorted (upper panel), revealing a stable line termed CAL-1 PH-Akt-GFP^line^. After 21 days in culture under G418 selection, two sub-lines were generated according to the gating strategy shown in the lower panel. **(B)** PH-Akt-GFP accumulates at the leading edge of migrating cells. R848-matured GM/CAL-1 PH-Akt-GFP^subline 2^ cells were embedded in 3D collagen and allowed to migrate towards CCL19. Bright field (upper left panel), GFP-fluorescent confocal (middle left panel), and inverted gray scale confocal (lower left panel) image of two individual migrating cells are shown. Line scan plots for PH-Akt-GFP along the cell axis of the two cells are depicted in the right panels. **(C)** Surface expression of CCR7 (left flow cytometry panel; blue solid line; isotype control: dashed line) and PH-Akt-GFP fluorescence (right flow cytometry panel) in R848-matured GM/CAL-1 PH-Akt-GFP^line^ cells was determined by flow cytometry. Maximal PH-Akt-GFP projection of a cell migrating through a 3D collagen I matrix along a CCL19 gradient is shown on the right. The corresponding time-lapse confocal microscopy video 1 can be found in the supplement. **(D)** CAL-1 PH-Akt-GFP (sub)lines retain their transgene and comparably induce CCR7 upon exposure to GM/CSF and maturation by R848. Representative CCR7/PH-Akt-GFP dot plots (upper panel) and quantification of all three independent experiments performed with parental GM/CAL-1 cells or GM/CAL-1 PH-Akt-GFP line and sublines, respectively, for CCR7 surface expression (lower left bar graph) and PH-Akt-GFP expression (lower right bar graph) are depicted as mean values ± SEM of median fluorescence intensities. **(E)** R848-matured GM/CAL-1, GM/CAL-1 PH-Akt-GFP^line^, GM/CAL-1 PH-Akt-GFP^subline 1^, or GM/CAL-1 PH-Akt-GFP^subline 2^ migrate in response to 30nM CCL19 or 30nM CCL21 in a 2D Transwell chemotaxis assay. Percentage of migrated cells was determined by flow cytometry after 3h of migration. Mean values ± SD of 3 independent experiments are shown.

Importantly, we set out to probe the tractability of expressing fluorescent reporter proteins in this human DC line. As a proof-of-concept, we established a CAL-1 cell line stably expressing the PH-domain of Akt fused to EGFP (PH-Akt-GFP), a biosensor for the major second messenger lipid phosphatidylinositol-3,4,5-trisphosphate (35), that is produced down-stream of G-protein activation upon chemokine stimulation (44, 46). To achieve this, we nucleofected CAL-1 cells with the fluorescent reporter plasmid and let the cells grow under selective antibiotic pressure. After two weeks, GFP-positive cells were bulk sorted to establish a stable PH-Akt-GFP expressing CAL-1 cell line (**Figure 5A**). R848-matured GM/CAL-1 PH-Akt-GFP^line^ cells expressed endogenously induced CCR7 on the cell surface together with transfected PH-Akt-GFP (**Figure 5C**). These cells were subsequently subjected to ibiTreat µ-slide chemotaxis chambers and allowed to migrate along a CCL19 gradient within a 3D collagen environment. PH-Akt-GFP was expressed in the cytosol of R848-matured GM/CAL-1 cells and accumulated at the leading edge while cells migrated in 3D collagen towards CCL19 (**Figure 5C** and **Supplementary Video 1**). To substantiate these findings, we established two additional CAL-1 sublines with different PH-Akt-GFP expression levels, referred to as subline 1 and subline 2 (**Figure 5A**). R848-matured GM/CAL-1 PH-Akt-GFP^subline 2^ cells were allowed to migrate towards CCL19 in 3D collagen. Drawing a line alongside the cell axis and plotting the intensity of GFP along this line clearly revealed accumulation of PH-Akt-GFP at the front of the migrating cells (**Figure 5B**). Side-by-side comparison of parental CAL-1 with the CAL-1 PH-Akt-GFP line and the two CAL-1 PH-Akt-GFP sublines revealed similar CCR7 induction upon sequential exposure to GM-CSF and R848- induced maturation (**Figure 5D**). Importantly, the GM/CAL-1 PH-Akt-GFP line and the two sublines robustly migrated in response to CCL19 and CCL21 as assessed in Transwell migration assays (**Figure 5E**).

**Figure 6:**
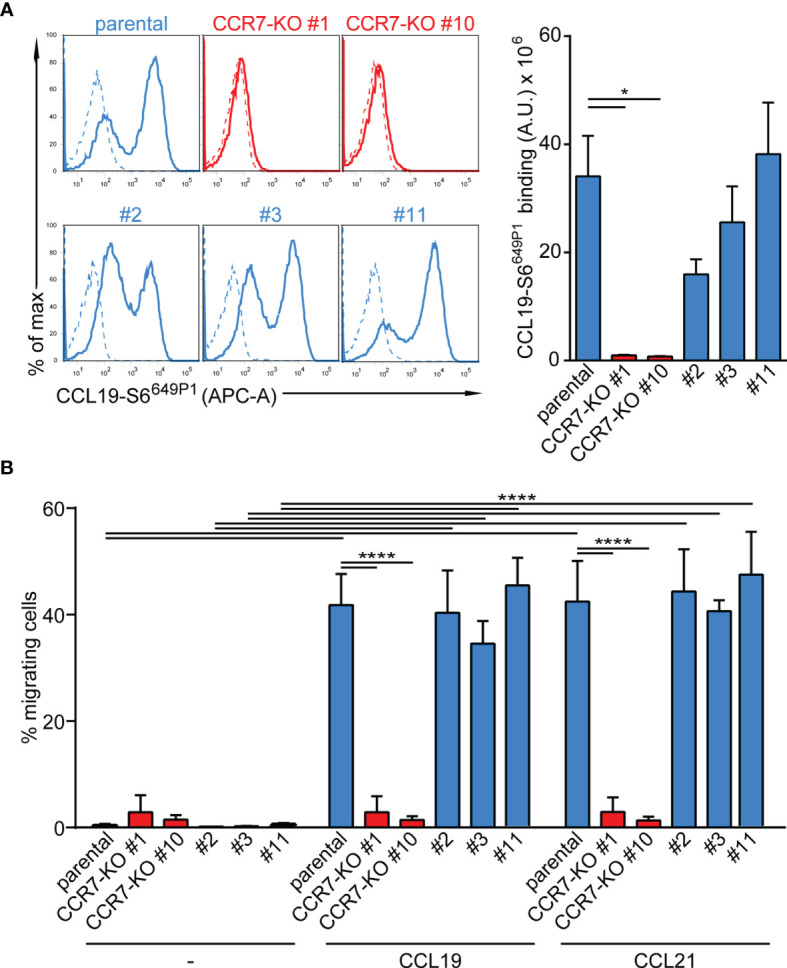
Functional characterization of CCR7-KO and parental CAL-1 single cell clones. **(A)** R848-matured GM/CAL-1 CCR7-KO single cell clones fail to bind fluorescent CCL19-S6^649P1^, while R848-matured parental GM/CAL-1 cells or individual parental single cell clones bind the fluorescent chemokine. Cells (1.8x 10^5^) were incubated for 30min with 20nM of fluorescent chemokine at 7°C. Chemokine binding was measured by flow cytometry. Representative histograms (solid lines: with chemokine, dashed lines: without chemokine) and quantification of CCL19-S6^649P1^ binding to R848-matured GM/CAL-1 cells (parental), GM/CAL-1 CCR7-KO clone #1 and #10, as well as three single clones (#2, #3, #11) established from parental CAL-1 cells. A representative histogram plot and mean values ± SEM of 3 independent experiments are shown. For quantification, the area under the curve [A.U.] was calculated by multiplying the mean CCL19-S6^649P1^ fluorescence intensity (MFI) values by the event count of live cells. **(B)** R848-matured GM/CAL-1 single cell clones retain their migratory capacity while CCR7-KO clones do not. R848-matured parental GM/CAL-1 and individual single cell clones were assessed by 2D Transwell migration assays in response to medium (–), 30nM CCL19 or CCL21 for 3h. Percentage of migrating cells was determined by flow cytometry. Mean values ± SD of 3 independent experiments are shown.

Finally, we targeted CCR7 by CRISPR/Cas9 gene editing to generate two CCR7-knockout CAL-1 cell clones, named CCR7-KO #1 and CCR7-KO #10. In addition, we established three CAL-1 clones (#2, #3, #11) from parental CAL-1 cells by single cell sorting. Parental CAL-1 cells, the two CCR7-KO clones and the three single cell clones were exposed to GM/CSF followed by R848-driven maturation. Parental GM/CAL-1 cells, as well as the three parental cell clones effectively bound fluorescently labelled CCL19-S6^649P1^ (41) although with some clonal variation (**Figure 6A**). By contrast, the CCR7-KO cell clones failed to bind the fluorescent CCR7 ligand (**Figure 6A**). Moreover, GM-CSF exposed and R848-matured CCR7-KO clones failed to migrate in response to CCL19 and CCL21, whereas the parental R848-matured GM/CAL-1 cell line and the individual cell clones efficiently migrated towards the chemokines (**Figure 6B**).

In summary, we demonstrated that CAL-1 cells induced CCR7 expression if exposed to GM-CSF and matured by the TLR7/8 ligand Resiquimod R848. To the best of our knowledge, this is the first report of a human DC-like cell line with endogenous CCR7 expression permitting to study CCR7 signaling and migration. As a proof-of-concept using PH-Akt-GFP, we further show that CAL-1 cells can be genetically modified to express fluorescent reporter proteins to study spatio-temporal signaling. The established CAL-1 PH-Akt-GFP cell lines and sublines remain stable and possess comparable migration capacities as the parental cell line. Moreover, we demonstrate that knockout cell clones can be generated. Hence, we here provide evidence that GM/CAL-1 cells serve a unique, valuable cellular model system to study human DC migration.

## Discussion

DCs are used as cellular vaccines to transport and deliver tumor antigens to cognate T cells to augment tumor antigen-specific T cell responses in cancer patients. Importantly, to be successful as cellular immunotherapy, DCs must migrate to lymph nodes to encounter antigen-specific T cells and to launch an adaptive immune response against the tumor (7). Hence, understanding how chemokines guide DC migration and homing to lymph nodes is pivotal, and cellular model systems to study human DC migration are highly desired. The chemokine receptor CCR7 is responsible for controlling DC migration from peripheral tissues to draining lymph nodes. Whereas most blood circulating T cells express CCR7, peripheral DCs induce this chemokine receptor only upon encounter of danger signals (13, 14, 26). CCR7 induction has been shown to predominantly involve the transcription factors NF-κB and AP-1 (47, 48). Interestingly, among all chemokine receptors, CCR7 possesses a unique signal sequence that facilitates its package into COPII vesicles for efficient ER to Golgi trafficking, and thus surface expression (36). Whereas bacterial-derived LPS, a ligand for TLR4, is the most used and effective danger signal to induce CCR7 expression on mouse BMDCs (49), human MoDCs only poorly respond to LPS, but instead readily respond to pro-inflammatory cytokines and synthetic TLR ligands, such as CpG or Resiquimod (13, 14, 26). MoDCs are likely to be the most commonly used model system to study human DCs. However, human MoDCs show substantial donor to donor variations and need to be differentiated from freshly isolated blood monocytes. A human cell line with DC characteristics and features that can be genetically manipulated would be a valuable tool to investigate molecular mechanisms controlling CCR7-depedent cell migration. Human leukemia cells of myeloid origin, such as conventional, as well as differentiated and matured HL60 and THP1 cells, or MUTZ cells were tested (29), but none of these attempts succeeded in cells that expressed CCR7 and migrated towards its chemokine ligands.

In the present study, we explored the human CAL-1 cell line that has been established from a patient with blastic plasmacytoid DC neoplasm (30). Although Karrich and colleagues previously reported a homogeneous CCR7 expression on CAL-1 cells in the absence of a maturation stimulus without assessing receptor functions (32), we did not detect CCR7 under these conditions. Moreover, in our hands, exposing CAL-1 cells to CpG-B failed to induce CCR7 expression. However, we observed that CAL-1 cells stimulated with Resiquimod slightly induced CCR7. Spurred by this observation, combined with previous reports that CAL-1 cells (in contrast to plasmacytoid DCs) are sensitive to GM-CSF (30, 34), we exposed CAL-1 cells to GM-CSF for 3 days and subsequently triggered them by synthetic TLR ligands. Exposure to GM-CSF resulted in the down-regulation of CD123, while enhancing the expression of CD11c and MHC class II. Moreover, GM/CAL-1 cells were able to take-up FITC-dextran, suggesting that they are able to take-up antigens, and to secrete cytokines like TNFα. Importantly, such GM/CAL-1 cells triggered with Resiquimod induced expression of endogenous CCR7. Hence, GM/CAL-1 cells share critical characteristics and features of human MoDCs. We further demonstrated that Resiquimod-matured GM/CAL-1 cells readily migrate in response to both CCR7 ligands, CCL19 and CCL21, in two-dimensional and 3-dimentional collagen environments. Moreover, CCR7 activation triggered, in a G_i_-protein dependent manner, a transient Ca^2+^ response in Resiquimod-matured GM/CAL-1 cells, which is a key component of the migration machinery (45, 50). Notably, in our hands, Resiquimod-stimulated CAL-1 cells that were not previously exposed to GM-CSF remained rather immobile and essentially failed to migrate towards CCR7 ligands in 3D environments.

Finally, the ability to genetically modify human DC-like cells is highly desired. As a proof-of-concept, we established a CAL-1 cell line and two sublines stably expressing the fluorescent biosensor PH-Akt-GFP, which upon exposure to GM-CSF and maturation with Resiquimod, accumulated at the leading edge of cells migrating towards CCR7 ligands. Interestingly, we also noted vesicular structures at the front of migrating cells that stained for PH-Akt-GPF. This observation is in line with our previous study showing endomembrane CCR7 signaling (43). Hence, CAL-1 cells expressing fluorescent biosensors can be exploited in future studies to shed light in spatial signaling during cell migration. Moreover, CAL-1 cells can be modified by introducing siRNA as successfully reported by Miura and colleagues (34). Here, we provide evidence that CAL-1 cells can also be gene edited using the CRISPR/Cas9 technology, thereby extending the palette of technologies to genetically modify and exploit this cell line. Taken together, in the present study, we demonstrated that human CAL-1 cells serve as versatile cellular model system to study CCR7-guided human DC migration.

## Data Availability Statement

The original contributions presented in the study are publicly available. This data can be found here: https://zenodo.org/record/4719596#.YUC2-J0zaUm and https://zenodo.org/record/5507215#.YUDCMp0zaUk.

## Author Contributions

EU-vA and DFL designed the study, analyzed data and wrote the manuscript. EU-vA, GS, and VP performed all experiments. TM provided essential reagents. All authors reviewed the manuscript. DFL supervised the overall study and acquired funding. All authors contributed to the article and approved the submitted version.

## Funding

This study was supported in parts by research funding from the Swiss National Science Foundation (grant number 310030_189144), the Thurgauische Stiftung für Wissenschaft und Forschung, and the State Secretariat for Education, Research and Innovation to DFL.

## Conflict of Interest

The authors declare that the research was conducted in the absence of any commercial or financial relationships that could be construed as a potential conflict of interest.

## Publisher’s Note

All claims expressed in this article are solely those of the authors and do not necessarily represent those of their affiliated organizations, or those of the publisher, the editors and the reviewers. Any product that may be evaluated in this article, or claim that may be made by its manufacturer, is not guaranteed or endorsed by the publisher.

## Supplementary Material

Supplementary Video 1 Representative time-lapse confocal microscopy video (Maximal projections; duration: 32min; scale bar: 10μm) of a R848-matured GM/CAL-1 PH-Akt-GFP^line^ cell migrating along a CCL19 gradient formed in a three-dimensional collagen I matrix using ibiTreat µ-slide chemotaxis chambers. The image depicted in **Figure 5C** is taken from this movie at t= 11min.
